# The Use of a Combination of RDC and Chiroptical Spectroscopy for Determination of the Absolute Configuration of Fusariumin A from the Fungus *Fusarium* sp.

**DOI:** 10.1007/s13659-015-0084-0

**Published:** 2016-01-20

**Authors:** Liang-Yan Liu, Han Sun, Christian Griesinger, Ji-Kai Liu

**Affiliations:** College of Agronomy and Biotechnology, Yunnan Agricultural University, Kunming, 650201 China; Department of NMR-based Structural Biology, Max-Planck-Institute of Biophysical Chemistry, Am Fassberg 11, 37077 Göttingen, Germany; Leibniz-Institut für Molekulare Pharmakologie, 13125 Berlin, Germany; School of Pharmaceutical Sciences, South-Central University for Nationalities, Wuhan, 430074 China

**Keywords:** NMR, Residual dipolar coupling, Relative configuration, Absolute configuration, Chiroptics

## Abstract

A new alkylpyrrole derivative, fusariumin A (**1**), was isolated from the culture broth of the fungus *Fusarium* sp. The absolute configuration of fuasiumin A has been established as (2′*R*,3′*R*) using a combination of RDC (residual dipolar coupling)-based NMR and DFT-supported chiroptical spectroscopy. It is worth to note that in this study without the aid of the RDC analysis, an unambiguous determination of configuration and conformation was not feasible due to the excessive conformational possibilities of this open-chain compound.

**Electronic supplementary material:**

The online version of this article (doi:10.1007/s13659-015-0084-0) contains supplementary material, which is available to authorized users.

*Fusarium* is a large genus of filamentous fungi which widely distributed in soil and associated with plants. Most species are harmless saprobes, and are relatively abundant members of the soil microbial community. Some species infest rice, maize, oats, barley, and wheat as pathogens and produce mycotoxins in cereal crops that can affect human and animal health if they enter the food chain [1]. The main toxins produced by these *Fusarium* species are fumonisins and trichothecenes. When contaminated food and feed are ingested, toxins initiate a wide range of acute and chronic symptoms, including cardiovascular lesions, hypotension, anemia and lymphoid necrosis [2, 3]. Chemically, *Fusarium* species are productive fungi which produce secondary metabolites with diverse structures. According to the structure types, compounds reported from *Fusarium* sp. can be divided into trichothecens, fumonisins, zearalenones, enniatins, butenolides, equisetins, and fusarins, respectively [4–7]. Some of these compounds exhibit notable biological activities. For instance, chlorofusin, a cyclic peptide isolated from the broth of *Fusarium* sp., showed significant antagonistic activity to the p53/MDM2 [8]. In this study, a new alkylpyrrole derivative was isolated from the culture broth of the fungus *Fusarium sp.*, and named it as fusariumin A (**1**, Fig. 1). The gross structure of **1** was established by extensive spectroscopic analysis, including MS, 1D and 2D NMR spectroscopy. Since the two stereocenters are located in a flexible alky chain, their relative configurations are difficult to be determined due to insufficient experimental data of scalar couplings and NOEs. Following the recent success in the determination of relative and absolute configuration of flexible and complex organic molecules by using a combination of residual dipolar coupling (RDC)-based NMR spectroscopy [9–15] and chiroptics [16–19], the complete stereochemistry and preferred conformation of fusariumin A has been determined. Our study here demonstrates again the power of RDC analysis in combination with chiroptics for the configurational and conformational analysis of challenging organic molecular systems.

**Fig. 1:**
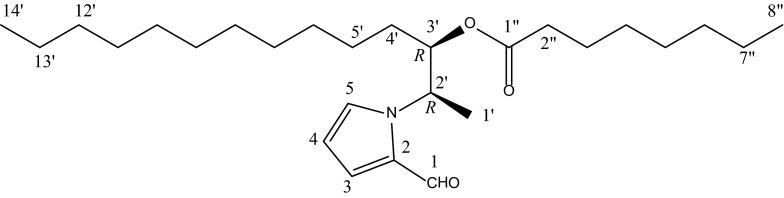
The structure of fusariumin A (**1**)

Fusariumin A was isolated as colorless oil with the molecular formula of C_27_H_47_NO_3_ which was deduced from the M^+^ ion at *m/z* 433.3556, corresponding to 5 degrees of unsaturation. One aldehyde proton at *δ*_H_ 9.53 (1H, s), and three aromatic protons at *δ*_H_ 6.92 (1H, dd, *J* = 4.3, 1.7 Hz), 6.22 (1H, dd, *J* = 4.3, 2.9 Hz) and 7.08 (1H, dd, *J* = 2.9, 1.7 Hz) in the down-field region of ^1^H NMR spectrum (CDCl_3_, Table S1, Electronic supplementary material), together with the carbons at *δ*_C_ 179.9 (d), 131.5 (s), 125.6 (d), 110.0 (d) and 127.7 (d) (CDCl_3_, Table S1) were empirically deduced to be a 1*H*-pyrrole-2-carbaldehyde moiety which was confirmed by the cross-peaks from H-5 to C-2, from H-1 to C-2 and C-3 in the HMBC experiment. In addition to the 1*H*-pyrrole-2-carbaldehyde moiety, the left 22 carbons involving one ester carbonyl, two methines, sixteen methylenes and three methyls required a linear structure to fulfil the degrees of unsaturation. The connection of C-1′/C-2′/C-3′/C-4′ was established from the correlations from H-1′ to C-2′ and C-3′, and from H-2′ to C-4′ in the HMBC spectrum (Fig. S2, Electronic supplementary material). C-2′ was deduced to be lined with the 1*H*-pyrrole-2-carbaldehyde moiety by the *N* atom from its chemical shift at *δ*_C_ 54.2 (d) and the HMBC correlation from H-2′ to C-2 and C-5. Similarly, the ester carbonyl group was determined at C-3′ by the chemical shift at *δ*_C_ 75.4 (d) and the HMBC correlation from H-3′ to C-1′′. Up to now, the core structure of compound **1** was determined and the remaining two methyls and sixteen methylenes required two side chains extending from C-3′ and C-1′′, respectively. In order to determine the length of each chain, high-resolution ESI-MS/MS technique was employed. When the [M + H]^+^ was selected for MS/MS analysis, the characteristic fragmentation ion at *m/z* 290.2483 with the chemical composition C_19_H_31_NO was detected, which could be explained by the elimination of a C_8_H_16_O_2_ part, namely an octanoic acid (Fig. S3, Electronic supplementary material). Therefore, the acyl at C-3′ was determined as an octanoyl group, and the left side chain extending from C-3′ was deduced with fourteen carbons (Table 1).

Fusaiumin A (Fig. 1) is a molecule that contains a large number of potentially rotatable bonds, leading to a complex conformational space that is difficult to sample. In this study, in order to simplify the conformational problem, only the conformations stemming from the rotation of the bonds C4′-C3′, C3′-C2′, and C2′-N, which are close to the two unknown stereocenters, were considered. This approach is further justified, since the long poly-methylene chains predominantly have antiperiplanar conformation, the NMR parameters except for RDCs are local and we have shown on at least two examples, i.e., sagittamide A [11] and fibrosterolsulfate A [14], that the single-tensor assumption is valid even if large parts of the molecule are not characterized in detail. Because of a small proton-proton coupling between H4′a and H3′ (around 3 Hz) and a large coupling between H4′b and H3′ (around 9 Hz), as shown in Table 2; Fig. S4 (Electronic supplementary material), only two out of three conformers of the C5′-C4′-C3′-O dihedral are possible main conformations. Since from the *J*-coupling analysis no conclusive results can be obtained regarding the major conformation of the C4′-C3′-C2′-N dihedral, all three conformations (C4′/N: +*gauche*,-*gauche*, *anti*) needed to be considered. Besides, two conformations for the H2′-C2′-N-C5 dihedral (H2′/C5: *trans*, *cis*) were found from DFT calculations. Taken together, 12 (= 2 × 3 × 2) conformations were generated and DFT optimized for the two possible relative configurations (2′*R*,3′*R*) and (2′*R*,3′*S*), respectively (Fig. S5; Table S2). For all these 12 conformations of each configuration, the alkyl chain from C4′ to C14′ and from C2′′ to C8′′ was assumed to adopt an *anti* conformation, which is the energetically most preferable conformer [20].

**Table 1:** ^1^H and ^13^C NMR spectroscopic data of fusariumin A (**1**) in DMSO at 700 MHz

No.	^1^H NMR	^13^C NMR
**1**	9.50, s	180.2, d
**2**		131.7, s
**3**	7.04, dd, 4.3, 1.7	126.0, d
**4**	6.24, dd, 4.3, 2.9	110.3, d
**5**	7.45, dd, 2.9, 1.7	129.6, d
**1**′	1.44, d, 7.2	15.1, q
**2**′	5.37, m	54.4, d
**3**′	5.03, m	75.1, d
**4**′**a** **4**′**b**	1.43, m 1.34, m	30.6, t
**5**′**a** **5**′**b**	1.22, m 1.16, m	25.2, m
**1**′′		172.6, s
**2**′′	2.24, m	33.9, t
**3**′′	1.46, m	24.9, t

**Table 2:** Long-range proton-proton and proton-carbon coupling constants of fusariumin A (**1**), *n* no signals in the HSQMBC and HMBC spectra

Atoms	^3^ *J*_HH_ [Hz]	Atoms	^2,3^ *J*_CH_ [Hz]
H3′-H4′a	3.3	H2′-C3′	−1.4
H3′-H4′b	9.4	H3′-C2′	−2.6
		H3′-C1′	3.0
		H3′-C5′	2.8
		H4′b-C2′	n
		H5′a-C3′	1.7
		H5′b-C3′	n
		H2′-C4′	n

After the attempt to determine the relative configuration of the two neighboring stereocenters C2′ and C3′ using ^2,3^*J*-couplings (Table 2) exclusively had failed, we decided to use residual dipolar couplings to probe the conformation and relative configuration of fusariumin A. Because only limited amount of sample (about 1 mg) was available, a slim PH-gel was prepared for a 1.7 mm NMR tube to acquire the RDC data [21]. 10 ^1^*D*_CH_ (Table S3, Electronic supplementary material) were extracted from the spectra and they were used to calculate the alignment tensor for each possible conformation using the SVD method [22]. In the RDC analysis, it was necessary to assign the two pairs of prochiral protons of C4′ and C5′, which has been achieved as follows: H5′a was defined as the proton that is closest to H3′ based on the strong NOE peak (Fig. S6); H4′b was defined as the proton which is *anti* to H3′ based on the large scalar couplings (Table 2; Fig. S4). The quality of the fit (*Q* factor) between the experimental RDCs and the back-calculated ones was used to identify the correct configuration and conformation [23]. The results summarized in Fig. 2 clearly show that the two lowest *Q* factors were obtained for conformations 9 and 11 of (2′*R*,3′*R*) or (2′*S*,3′*S*) with *Q* factors of 0.20 and 0.09, whereas the *Q* factors of all other possible conformers of (2′*R*,3′*S*) or (2′*S*,3′*R*) are significantly higher (Figs. S7, S8, Electronic supplementary material). Furthermore, as shown in Table S3 and Figure S8 (Electronic supplementary material), no significant violation of the experimental data is observed for any RDC of the best fitting conformer 11 of the relative configuration (2′*R*,3′*R*) or its enantiomer.

**Fig. 2:**
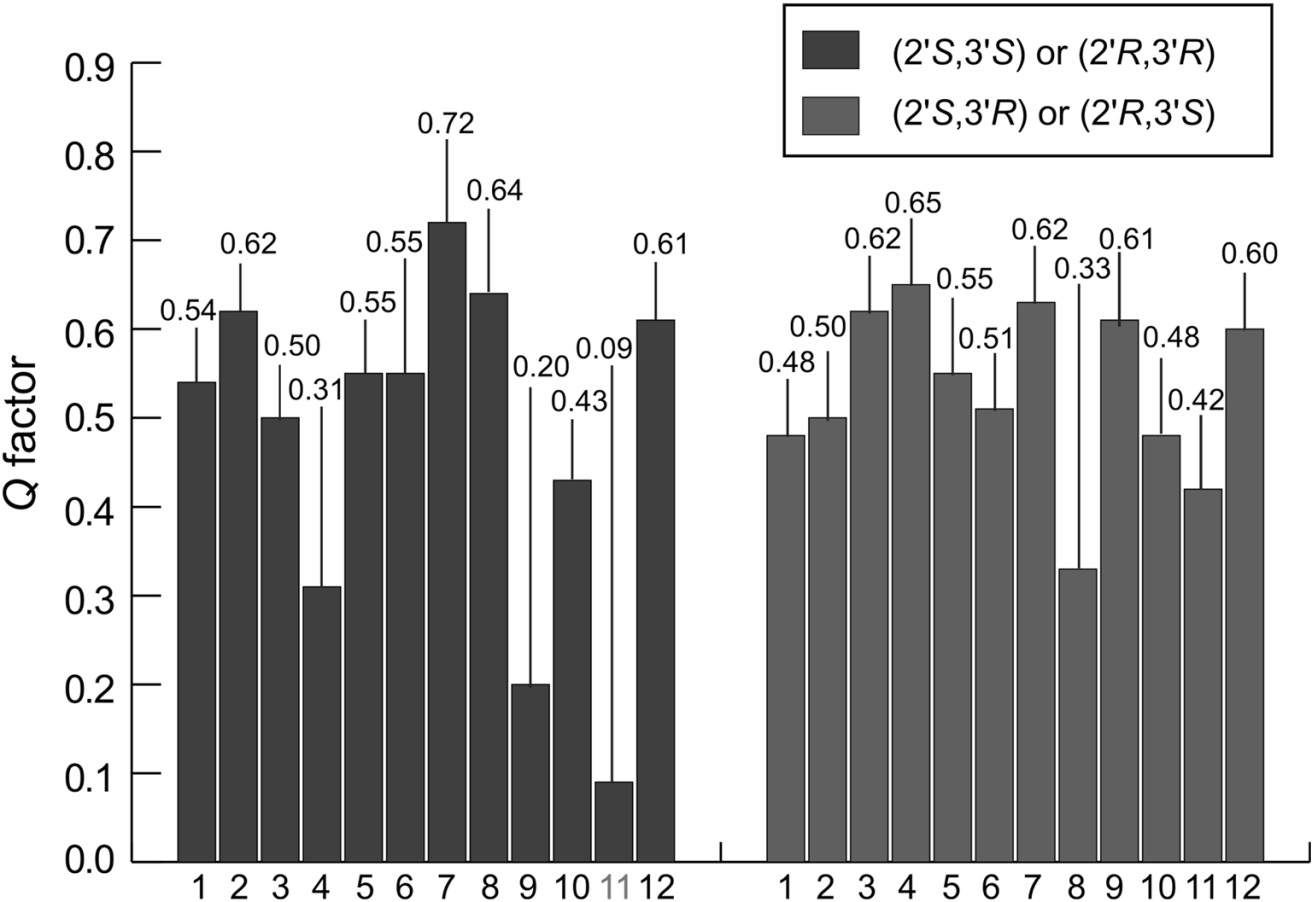
Comparison of the *Q* factors of the RDC fitting for the two possible relative configurations (2′*R*,3′*R* *black*) and (2′*R*,3′*S* *grey*) or their enantiomers. For each configuration 12 different conformers were considered in the RDC fitting. The conformer 11 of the configuration (2′*R*,3′*R*) or (2′*S*,3′*S*), which exhibits the significantly lowest *Q* factor, is highlighted in bold

Conformations 9 and 11 that exhibit the lowest *Q* factor of the RDC fitting (Fig. 2) mainly differ in the dihedral H3′-C3′-C2′-H2′. According to pervious studies [24, 25], for dihedral angles O-CA-CB-H, where CB bears one oxygen or nitrogen, the observed ^2^*J* values range from -6 to 0 Hz. In addition, the *anti* conformation between proton and heteroatom leads to a small value of the two bonds C/H coupling constant (^2^*J*_CH_), whereas a large negative coupling constant is associated with a *gauche* conformation. Due to the fact that small negative coupling constants for both H2′/C3′ and H3′/C2′, and a small positive coupling for H3′/C1′ (Table 2) were observed, the *anti* relationship between H3′ and N, as well as between H2′ and O was determined as the main conformation. This is the case for conformer 11, but not for conformer 9, as shown with the aid of the Newman projections in Fig. 3. Additionally, NOE peaks that are relevant in the configurational and conformational analysis have been integrated (Table S4, Electronic supplementary material). The relationship between NOE integrals and the distance to the power of minus six is in a good agreement for the following proton pairs: H3′ and H5′a, H1′ and H5, H3 and H1, H4′b and H3, H4′a and H3, H2′and H1, which further supports conformer 11 as the major conformer. However, a relatively smaller NOE integral of H5′b and H2′, together with a relatively larger NOE integral of H5′a and H2′, were observed compared to the predicted ones. The reason for this could be a conformational averaging around the dihedral C4′-C5′, which nevertheless does not change the conclusion that conformer 11 was determined as the main conformation, because only dihedrals of C3′-C4′, C2′-C3′ and C2′-N define the conformation for the configurational analysis of C2′ and C3′.

**Fig. 3:**
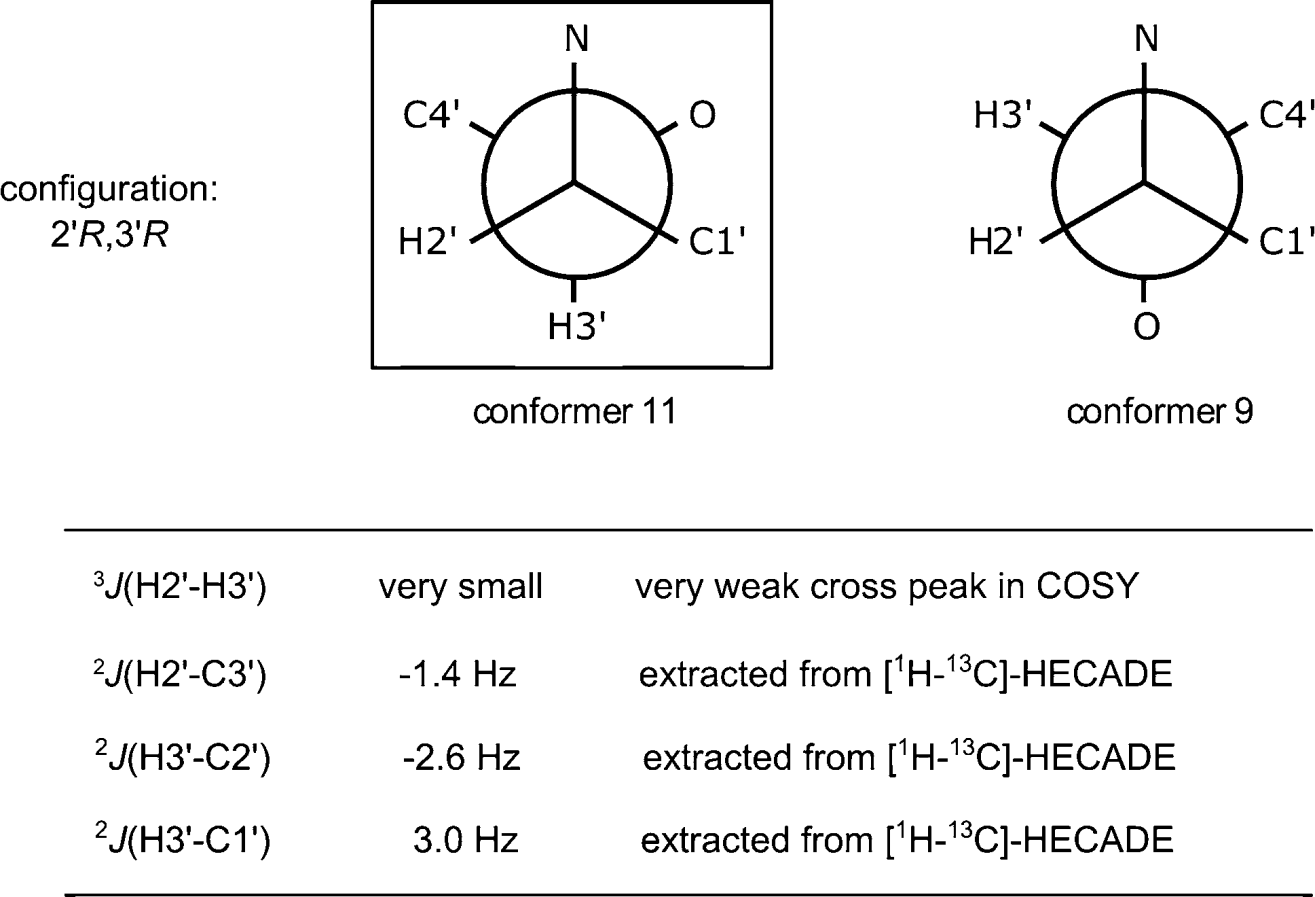
Newman projections of the conformers 9 and 11 for dihedral C2′-C3′. All conformers have a configuration of (2′*R*,3′*R*)

The energy and free energy of each possible conformer of the relative configuration (2′*R*,3′*R*) or (2′*S*,3′*S*) was calculated by DFT using the B3LYP/6-31G(d) basis set that has been employed in the structure optimizations as well. Comparison of the computational energy of individual conformers (Table S5, Supporting Information) identifies the conformer 2 (**Δ**E = 0, **Δ**G = 0) and NMR-determined conformer 11 (**Δ**E = 0.5 kcal mol^−1^, **Δ**G = 1.1 kcal mol^−1^) as the lowest energy conformations. However, due to bad agreement with the RDC data (*Q* = 0.62) as well as NOE violations, as the expected NOE between H2′ and H5 cannot be observed in the spectrum, the energetically most preferential conformation (conformer 2, see Table S2; Fig. S5) can be excluded to be the major conformation. Although many studies show that the NMR data are corroborated by DFT-based energy calculations [26, 27], on our example the DFT computation with the B3LYP functional do not accurately predict the relative energies [28].

In short, the relative configuration of fusariumin A was established as (2′*R*,3′*R*) or (2′*S*,3′*S*) by the RDC analysis exclusively. Furthermore, the preferential population of a single local conformation around the dihedrals of C3′-C4′, C2′-C3′ and C2′-N (conformer 11) was determined by the excellent fit of the RDC data, and was further corroborated by the *J*-coupling and NOE analysis. The long alkyl side chains were not analysed further, since they are assumed to be predominantly all-*trans* and were shown to have little influence on the RDC enhanced NMR analysis presented here [11, 14].

To determine the absolute configuration, ECD spectra of both enantiomeric forms (2′*R*,3′*R*) and (2′*S*,3′*S*) of NMR-determined conformation 11 have been calculated with time-dependent density functional theory (TD-DFT). The ECD spectrum calculated for (2′*R*,3′*R*) shown in Fig. 4 reproduced both the signs and the shape of the measured one. The agreement between the recorded spectrum and the calculated one is excellent, when the calculated spectrum of (2′*R*,3′*R*) is red-shifted by 25 nm. This procedure is in general allowed, because it has been shown in many previous studies that using the TD-DFT calculation with the B3LYP functionals the excitation energy can be predicted either too high or too low, depending on which system class is taken into account [29]. Based on the ECD data, the absolute configuration of fusariumin A was assigned as (2′*R*,3′*R*).

**Fig. 4:**
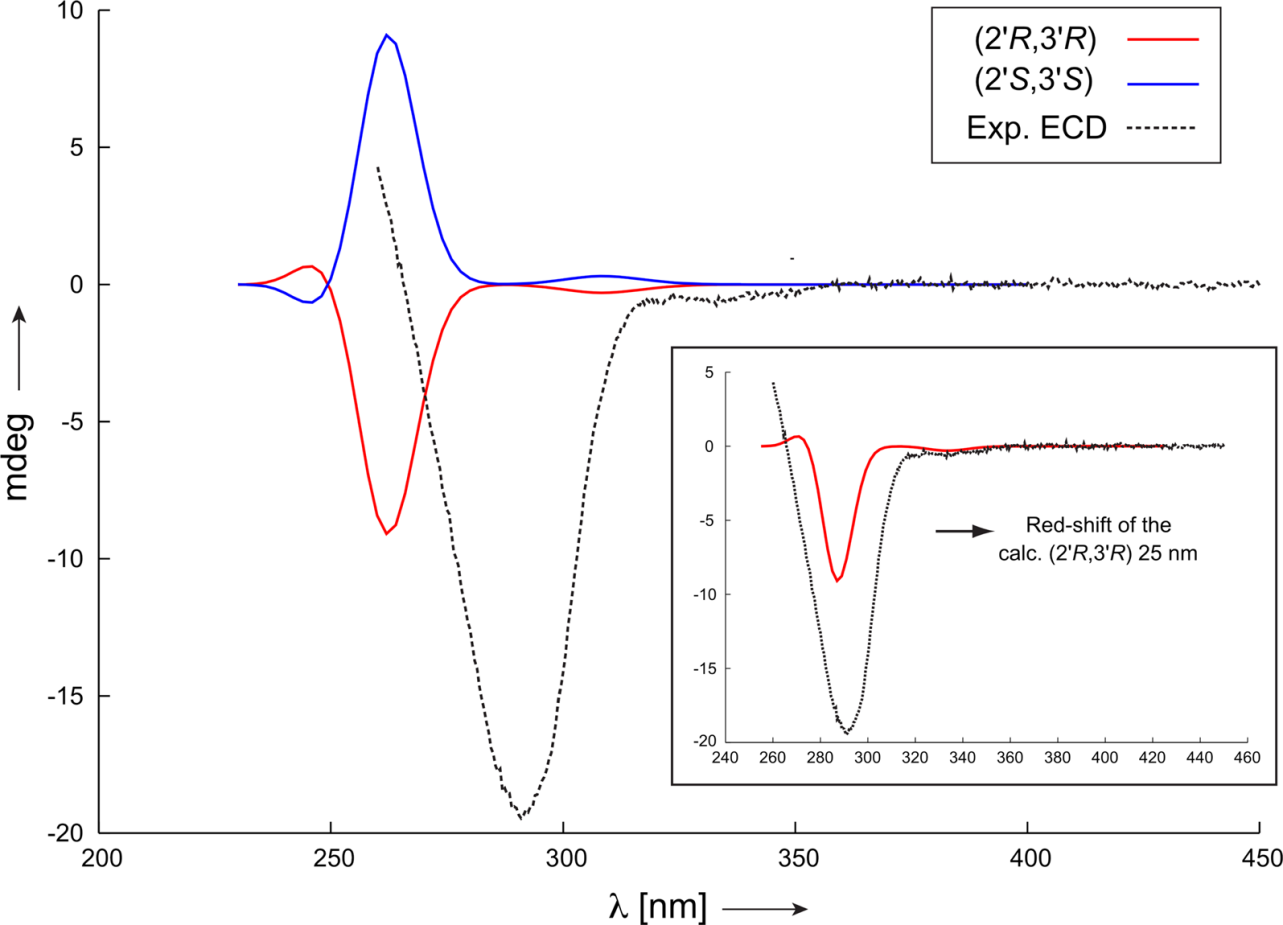
Comparison of experimentally measured ECD spectrum of fusariumin A in DMSO (*dashed line*) to the calculated ones using the RDC determined conformer 11 for both enantiomers (2′*R*,3′*R*) and (2′*S*,3′*S*). A 25 nm shift to higher wavelength of the calculated ECD spectrum of (2′*R*,3′*R*) results in a good fit to the experimental spectrum

Furthermore we measured ORD values at five different wavelengths (365, 436, 546, 578, 589 nm) in DMSO for fusariumin A, and calculated them for the conformer 11 with DFT. Figure 5 shows that the calculated ORD curve of (2′*R*,3′*R*) has a excellent agreement with the experimental one, which further supports the assignment of the absolute configuration from the ECD data. Interestingly, conformer 2 which was found by DFT to have the lowest energy shows an opposite sign of ECD and ORD as the ones of conformer 11 (Figs. S9, S10, Electronic supplementary material). Thus, without the NMR spectroscopic investigation which ruled out conformer 2 to be present in the solution ensemble, the wrong absolute configuration would have been determined. This result agrees with a previous finding [17] and further supports that the accurate determination of the conformation by RCD-based NMR analysis is essential for the correct assignment of absolute configuration for fusariumin A.

**Fig. 5:**
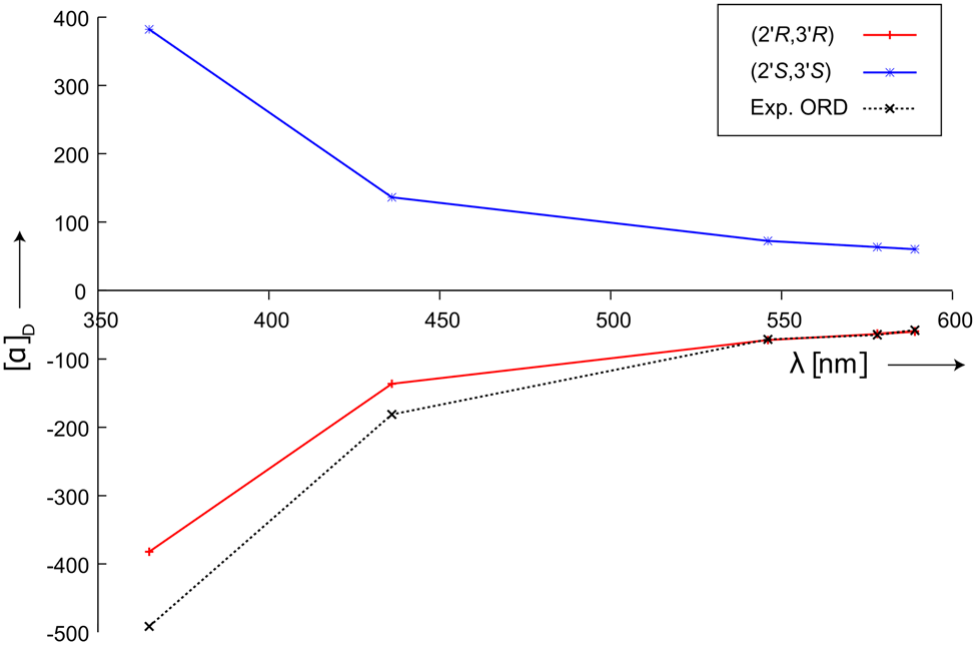
Comparison of experimentally measured ORD spectrum of fusariumin A in DMSO (*dashed line*) to the calculated ones using the RDC determined conformer 11 for both enantiomers (2′*R*,3′*R*) and (2′*S*,3′*S*)

In conclusion, the absolute configuration of fusariumin A has been established as (2′*R*,3′*R*) using a combination of NMR and DFT-supported chiroptical spectroscopy. It is worth to note that in this study without the aid of the RDC analysis, an unambiguous determination of configuration and conformation was not feasible due to the excessive conformational possibilities of this open-chain compound. Using the SVD fitting of the ^1^*D*_CH_ data on the individual possible conformers, not only the relative configuration was successfully established, but also the selection of the correct conformations has been remarkably simplified, which is the basis for the DFT calculation of the chiroptical properties. A NOE and *J*-coupling analysis was subsequently utilized to validate the RDC-determined conformation and configuration. To our surprise, prevalence of one conformer around the dihedral of C3′-C4′, C2′-C3′ and C2′-N has been determined in solution. Of course, there will be the normal conformational flexibility in the long alkyl chains with predominance of the all-*trans* conformation. According to the previous study [30], it is interesting to note that preferences with high weight for a specific conformation have been already observed for numerous open-chain polyketides, especially when the alkyl chains contain certain functional groups. For example, the methyl groups in the alkyl chains do not affect the flexibility of the backbone, nevertheless they reduce the number of low energy local conformers. Our study here shows that fusariumin A is another case, in which nature has chosen an acyclic backbone with a high preference to populate a preferred local conformation.

## Experimental Section

### General Experimental Procedures

Optical rotations were measured on a Horiba SEPA-300 polarimeter (Horiba, Tokyo, Japan). Optical rotations for the determination of absolute configuration were measured at 22–25 °C using a 2 mL cuvette at 589, 578, 546, 436 and 365 nm with a Perkin Elmer model 241 polarimeter (Waltham, Massachusetts, USA). IR spectra were obtained on a Bruker Tensor 27 FT-IR spectrometer (Bruker, Ettlingen, Germany) using KBr pellets. CD spectra were recorded at room temperature using a Jasco-J-810 CD spectrometer (Tokyo, Japan). Standard 1D and 2D NMR spectra of fusariumin A in CDCl_3_ were acquired on Bruker DRX-500 and AM-400 instruments at room temperature with TMS as internal standard (Bruker, Rheinstetten, Germany).Standard 2D NMR spectra with DMSO solvents, together with [^1^H,^13^C]-CLIP-HSQC [31], [^1^H,^13^C]-HECADE [32], [^1^H,^13^C]-HSQMBC [33] and NOESY [34] spectra, which were used for determination of relative configuration, were recorded on Bruker 700 MHz with 1.7 mm PA-TXI room temperature probe head (Bruker, Rheinstetten, Germany). Chemical shifts (*δ*) were expressed in ppm with reference to the solvent signals. Mass spectra (MS) were recorded on an API QSTAR time-of-flight spectrometer (MDS Sciex, Ontario, Canada) or a VG Autospec-3000 spectrometer (VG, Manchester, England). Silica gel (200–300 mesh, Qingdao Marine Chemical Inc., Qingdao, China), Sephadex LH-20 (Amersham Biosciences, Sweden), and RP-18 gel (40–75 μm, Fuji Silysia Chemical Ltd. Japan) were used for column chromatography (CC). Preparative HPLC (Prep-HPLC) was performed on an Agilent 1200 liquid chromatography system equipped with a ZorbaxSB-C_18_ column (9.4 mm × 150 mm). Pre-coated silica gel GF254 plates (Qingdao Marine Chemical Inc., Qingdao, China) were used for TLC. Fractions were monitored by TLC, and spots were visualized by heating silica gel plates sprayed with 10% H_2_SO_4_ in ethanol.

### Fungal Material

The fungus *Fusarium* sp. was collected in Botanic Garden of Kunming Institute of Botany, Chinese Academy of Sciences and identified by Prof. Zhu-Liang Yang (Kunming Institute of Botany, Chinese Academy of Sciences). A voucher specimen has been deposited in the School of Pharmaceutical Sciences, South-Central University for Nationalities. The culture medium consists of potato (peeled, 200 g), glucose (20 g), aneurine hydrochloride (10 mg), KH_2_PO_4_ (3 g), and MgSO_4_ (1.5 g) in deionized water (1 L). The pH was adjusted to 6.5 before autoclaving, and the fermentation was carried out in a shaker (150 rpm) at 25 °C for 25 days.

### Extraction and Isolation

The culture broth (18 L) was extracted with EtOAc for three times, and the organic layer was concentrated under reduced pressure to give a crude extract (8.0 g), which was subjected to silica gel column chromatography (CC) using a petroleum ether–acetone gradient (1:0 → 0:1) to afford fractions A–F. The fraction A was chromatographed first on a silica gel column eluted with petroleum ether–acetone (50:1, v/v), and then on a Sephadex LH-20 column eluted with CHCl_3_-MeOH (1:1) to afford two fractions A1 and A2. The fraction A1 was purified by Preparative HPLC (CH_3_CN/H_2_O, 60:40 → 100:0) to give fusariumin A (3.0 mg).

*Fusariumin A (1)*. colorless oil; [α]_D_^21^ = −108.6 (*c* 0.19, CHCl_3_); IR (KBr) *ν*_max_ 2926, 2855, 1741, 1668, 1467, 1164, 776 cm^−1^; ^1^H NMR (500 MHz, CDCl_3_) *δ*: 9.53 (1H, s, C*H*O), 7.08 (1H, dd, *J* = 2.9, 1.7 Hz, H-23), 6.92 (1H, dd, *J* = 4.3, 1.7 Hz, H-25), 6.22 (1H, dd, *J* = 4.3, 2.9 Hz, H-24), 5.55 (1H, m, H-21), 5.11 (1H, m, H-12), 2.23 (2H, m, H-14), 1.56 (4H, m, H-11 and H-15), 1.46 (3H, d, *J* = 7.1 Hz, H-22), 1.23 (26H, H-2 ~ H-10 and H-16 ~ H-19), 0.88 (6H, m, H-1 and H-20); ^13^C NMR (125 MHz, CDCl_3_) *δ*: 179.9 (d, *C*HO), 172.9 (s, C-13), 131.5 (s, C-26), 127.7 (d, C-23), 125.6 (d, C-25), 110.0 (d, C-24), 75.4 (d, C-12), 54.2 (d, C-21), 34.2 (t, C-14), 31.9 ~ 22.6 (C-2 ~ C-11 and C-15 ~ C-19), 14.7 (q, C-22), 14.1 (q, C-1 and C-20); ESIMS (positive): *m/z* 456 [M + Na]^+^, 890 [2 M + Na + H]^+^, 434 [M + H]^+^, 290; HREIMS: *m/z* 433.3564 (calcd. for C_27_H_47_NO_3_, 433.3556); 290.2473 (calcd. for C_19_H_32_NO, 290.2483).

### Preparation of the PH-gel for 1.7 mm NMR Tube

2-(Acrylamido)-2-methyl- propanesulfonic acid (1 M), *N,N*-dimethylacrylamide (1 M), *N,N***-**methylenebisacryl- amide (0.03 M), and ammonium persulfate (8 mM) were dissolved in purified water followed by devolatilization, in vacuo, for 15 min. The stock solution was inserted into a gel cylinder (material: PEEK) with an inner diameter of 2 mm and polymerized for 8–9 min at 70 °C. The gels were washed twice with aqueous HCl (0.02 M), three times with purified water (each time for 1–2 h) and finally dried under ambient conditions (air **+** room temperature) for at least 1 day.

### Molecular Mechanics and DFT Calculations

The initial structure of each possible conformation was built with Discovery Studio 2.5 (Accelrys) and all trial structures were first minimized based on molecular mechanics calculations (CFF force field) [35] and followed by DFT optimizations at B3LYP/6-31G (d) levels. DFT optimizations were performed by using Gaussian09 [36] by using the IEFPCM solvent continuum model with DMSO as the solvent.

### RDC Fitting

The fitting procedure of the experimental RDC data was performed by using the MSpin program (Mestrelab Research) [37].10 experimental ^1^*D*_CH_ couplings and the coordinate files obtained from DFT optimizations were given as the input data. Five independent members of the alignment tensor were determined by using the singular value decomposition (SVD) method. The goodness of fit between experimental and back-calculated RDCs was expressed in terms of the Cornilescu quality factor *Q* [23].

### ECD Computations

Time-dependent DFT (TD-DFT) with the basis set B3LYP/6-31G(d) was used to calculate the spin-allowed excitation energies, rotatory and oscillator strengths of the lowest 50 excited states. The calculations were performed with Gaussian09 [36] by using the IEFPCM solvent continuum model with DMSO as the solvent.

### ORD Computations

The optical rotation dispersion calculations were performed at the four wavelengths 365, 436, 546, 578 and 589 nm by using the optimized structures as input coordinates. The calculations were carried out with the basis set B3LYP/6-31G(d) by using the IEFPCM solvent continuum model as implemented in Gaussian09^36^ with DMSO as the solvent.

## Electronic supplementary material

Supplementary material 1 (DOCX 2068 kb)
