# Supplementary material for: The Use of a Combination of RDC and Chiroptical Spectroscopy for Determination of the Absolute Configuration of Fusariumin A from the Fungus Fusarium sp
Source: Nat Prod Bioprospect. 2016 Jan 20;6(1):41–8. doi: 10.1007/s13659-015-0084-0 (PMC4749523; doi:10.1007/s13659-015-0084-0)
Supplement: Supplementary file 1 — Supplementary material 1 (DOCX 2068 kb) [file 13659_2015_84_MOESM1_ESM.docx]

**Supporting Information**

**The Use of a Combination of RDC and Chiroptical Spectroscopy for Determination of the Absolute Configuration of Fusariumin A from the Fungus *Fusarium sp.***

Liang-Yan Liu, ^#,‡^ Han Sun, ^#,§,||^ Christian Griesinger, ^§,^* Ji-Kai Liu^†,^*

*^†^School of Pharmaceutical Sciences, South-Central University for Nationalities, Wuhan 430074, China*

*^‡^College of Agronomy and Biotechnology, Yunnan Agricultural University, Kunming 650201, China*

*^§^Max-Planck-Institute of Biophysical Chemistry, Department of NMR-based Structural Biology, Am Fassberg 11, 37077 Göttingen, Germany*

*^||^Leibniz-Institut für Molekulare Pharmakologie, 13125 Berlin, Germany*

*^#^Contributed equally to the work.*

**To whom correspondence may be addressed. Email,* [*cigr@nmr.mpibpc.mpg.de*](mailto:cigr@nmr.mpibpc.mpg.de) *or* [*jkliu@mail.kib.ac.cn*](mailto:jkliu@mail.kib.ac.cn)

^1^H NMR spectrum


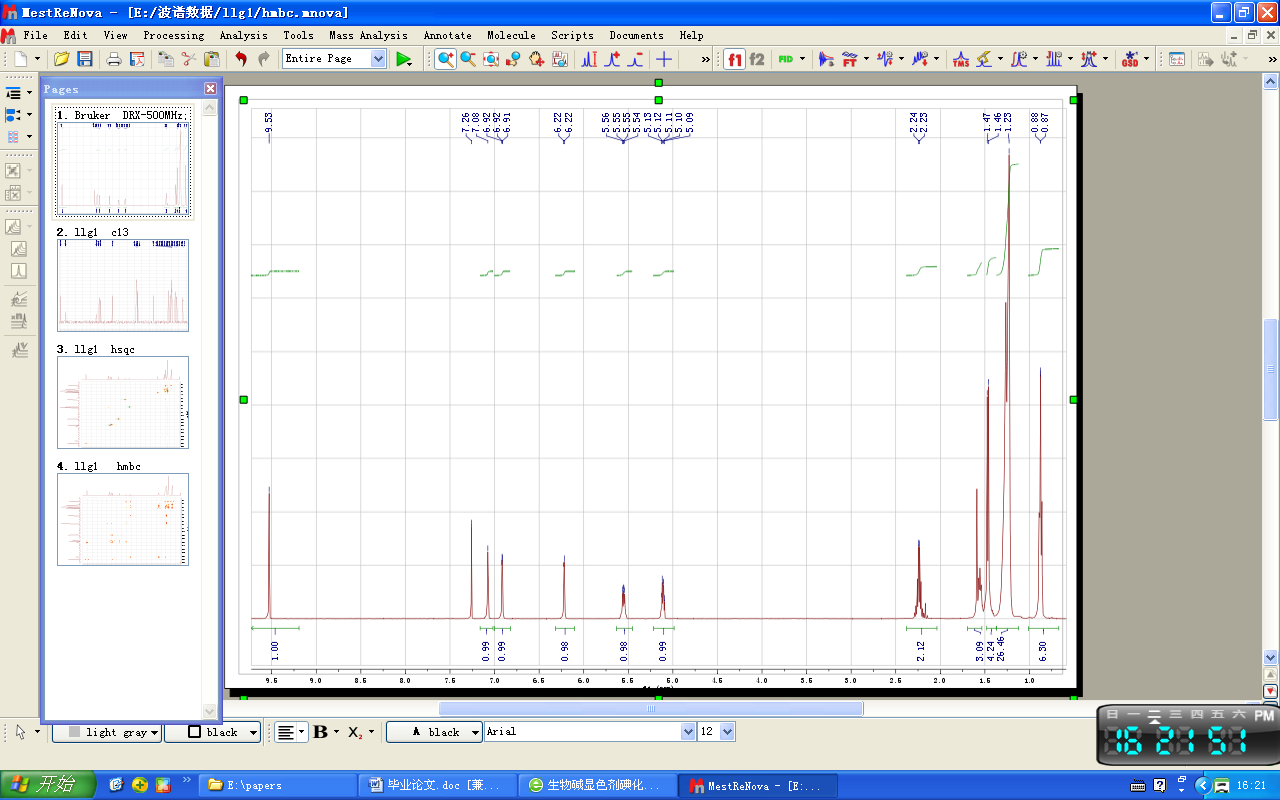


^13^C NMR spectrum


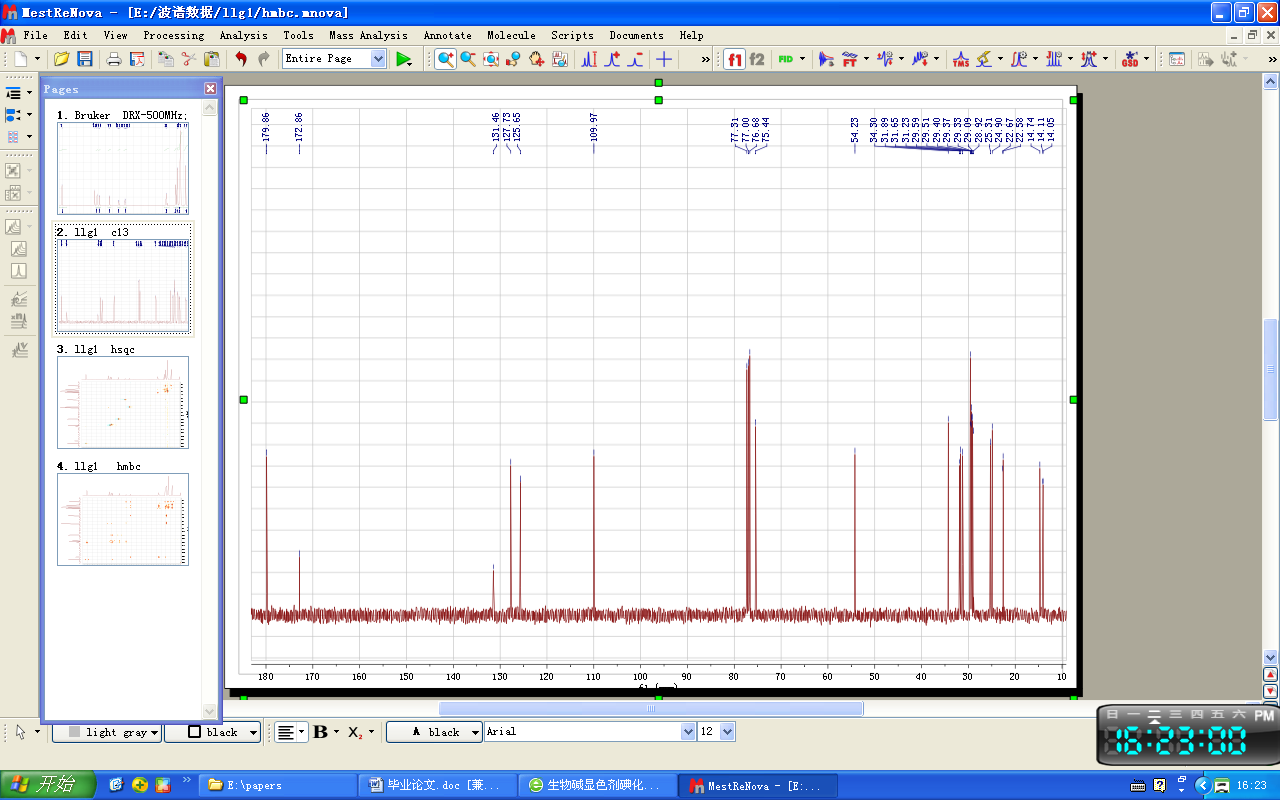


HSQC


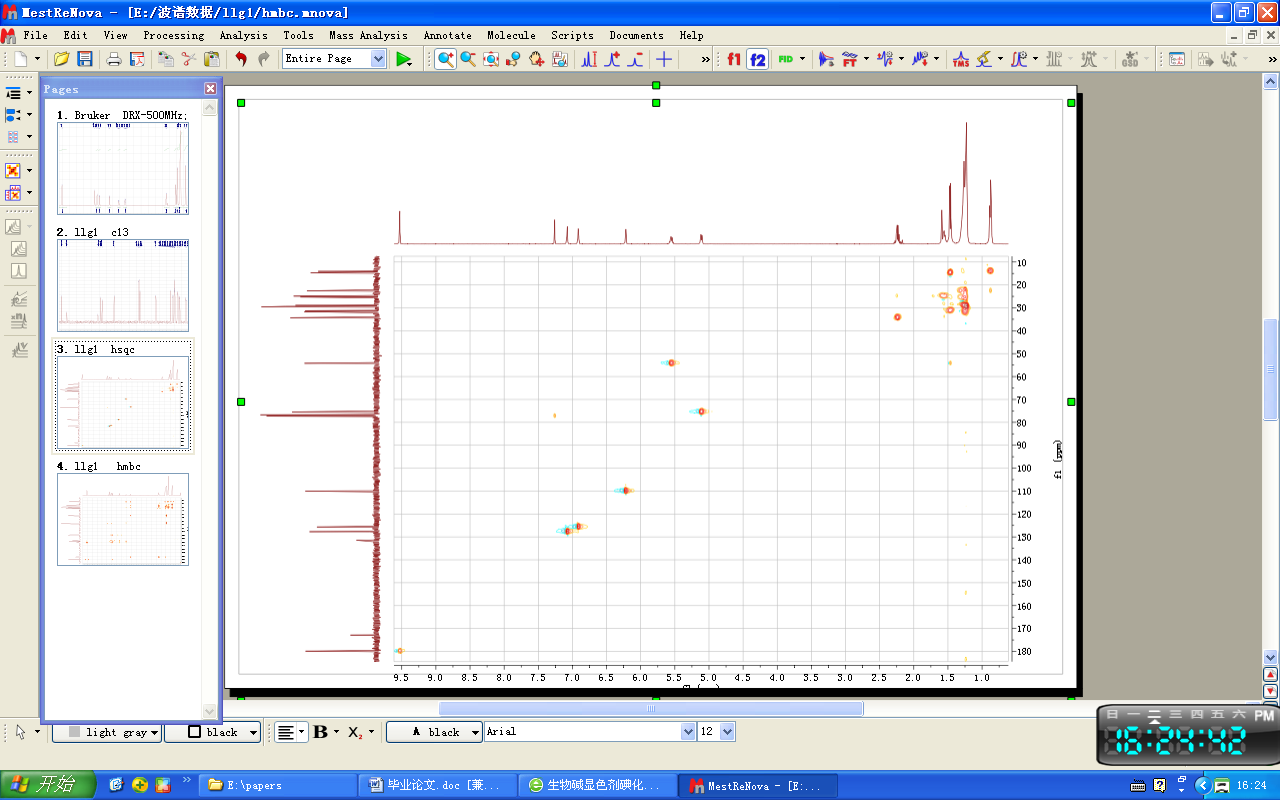


HMBC


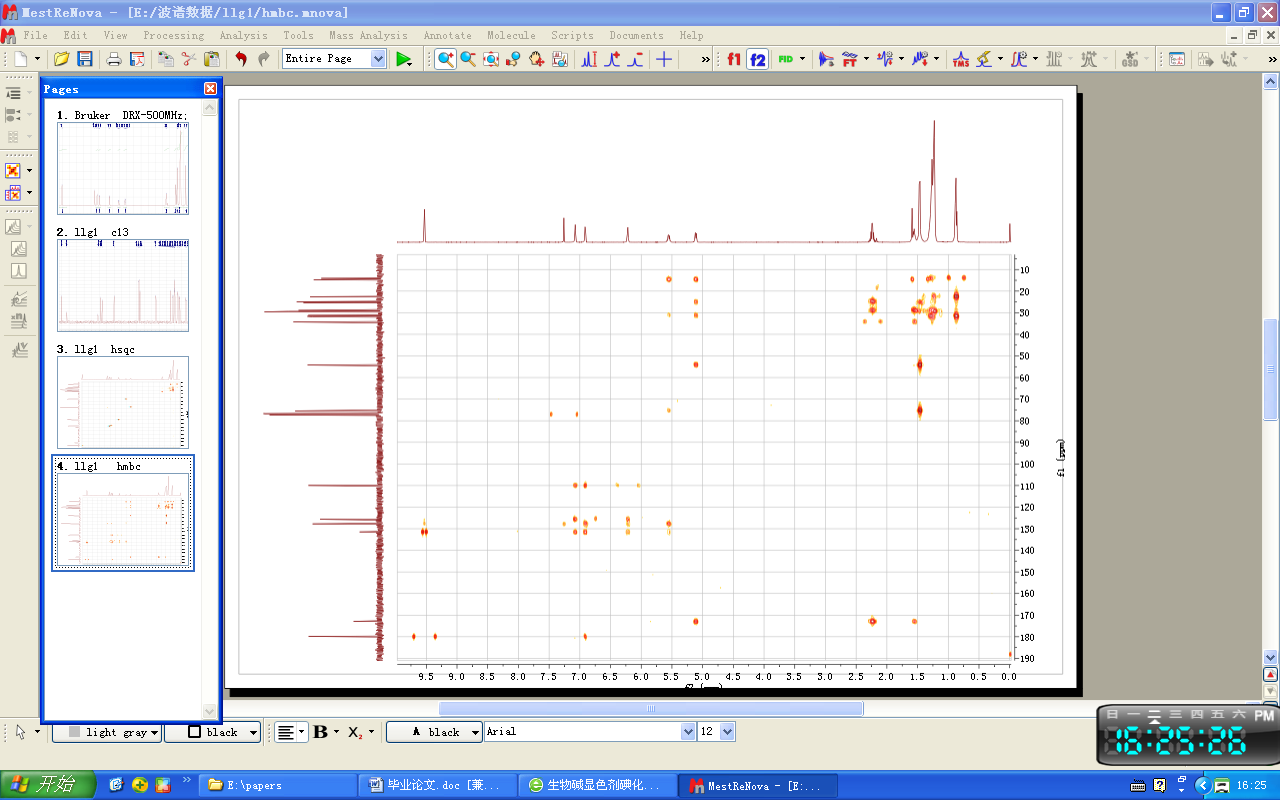


**Figure S1.** Standard 1D and 2D NMR spectra of fusariumin A in CDCl_3_ at 500 MHz.

**Table S1.** ^1^H and ^13^C NMR spectroscopic data of fusariumin A in CDCl_3_ at 500 MHz

| **No.** | ^1^H NMR | ^13^C NMR |
| --- | --- | --- |
| **1** | 9.53, s | 179.9, d |
| **2** |  | 131.5, s |
| **3** | 6.92, dd, 4.3, 1.7 | 125.6, d |
| **4** | 6.22, dd, 4.3, 2.9 | 110.0, d |
| **5** | 7.08, dd, 2.9, 1.7 | 127.7, d |
| **1'** | 1.46, d, 7.2 | 14.7, q |
| **2'** | 5.55, m | 54.2, d |
| **3'** | 5.11, m | 75.4, d |
| **4'** | 1.56, m | 25.3, t |
| **5'-11'** | 1.23, brs | 28.9-29.6, t |
| **12'** | 1.23, brs | 31.7, t |
| **13'** | 1.23, brs | 22.6, t |
| **14'** | 0.87, t, 6.8 | 14.1, q |
| **1''** |  | 172.9, s |
| **2''** | 2.23, m | 34.2, t |
| **3''** | 1.56, m | 24.9, t |
| **4''** | 1.23, brs | 29.6, t |
| **5''** | 1.23, brs | 29.4, t |
| **6''** | 1.23, brs | 31.9, t |
| **7''** | 1.23, brs | 22.7, t |
| **8''** | 0.88, t, 6.8 | 14.1, q |

**Figure S2.** The selected HMBC correlations of fusariumin A.

**Figure S3.** The MS/MS fragmentation of fusariumin A.


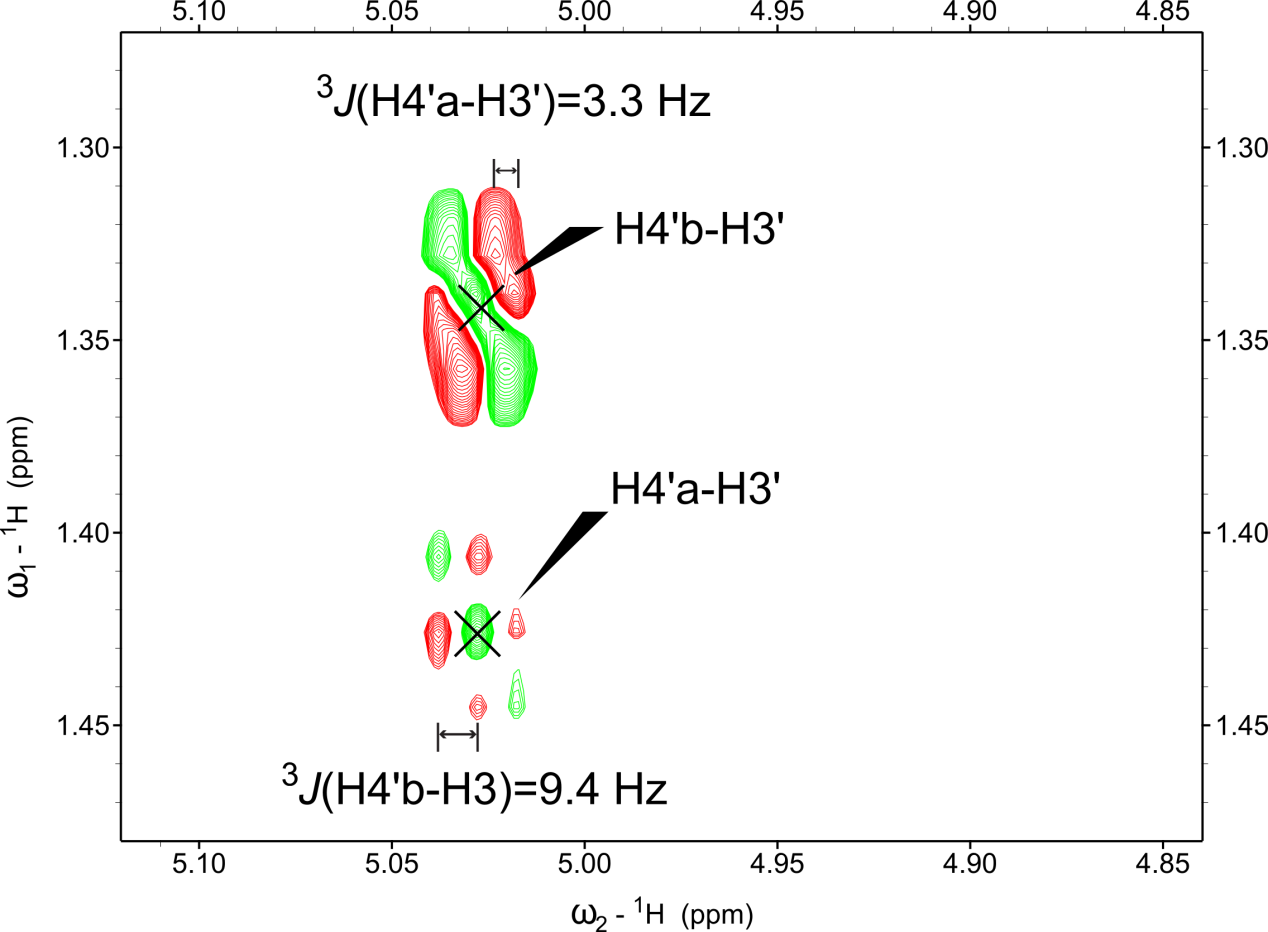


**Figure S4.** Regions from P.E.COSY spectrum showing the homonuclear couplings of H4'a-H3' and H4'b-H3'.


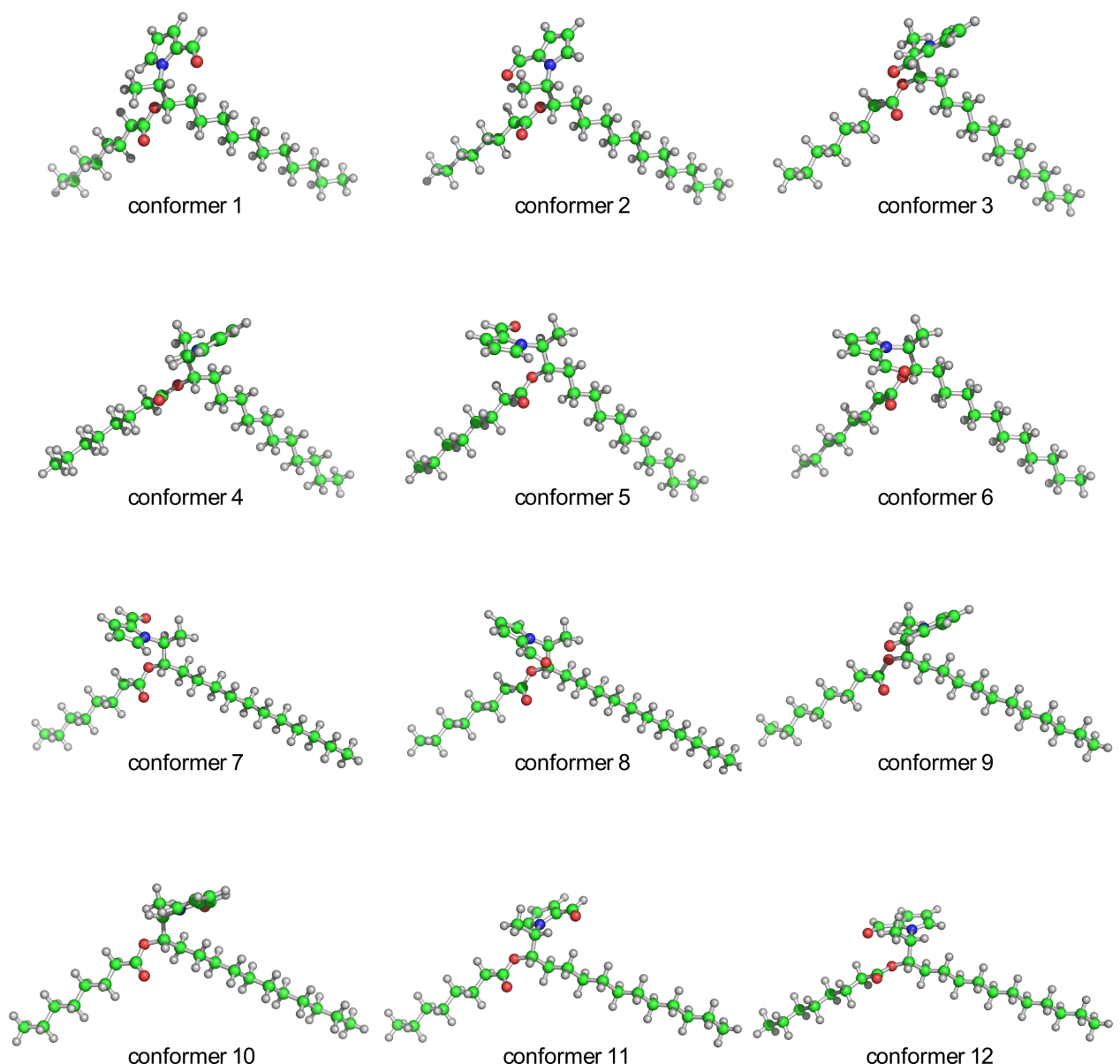


**Figure S5.** 12 possible conformers of fusariumin A with the configuration of (2'*R*,3'*R*). All structures were optimized at the B3LYP/6-31G(d) level using the IEFPCM solvent continuum model with DMSO as the solvent.


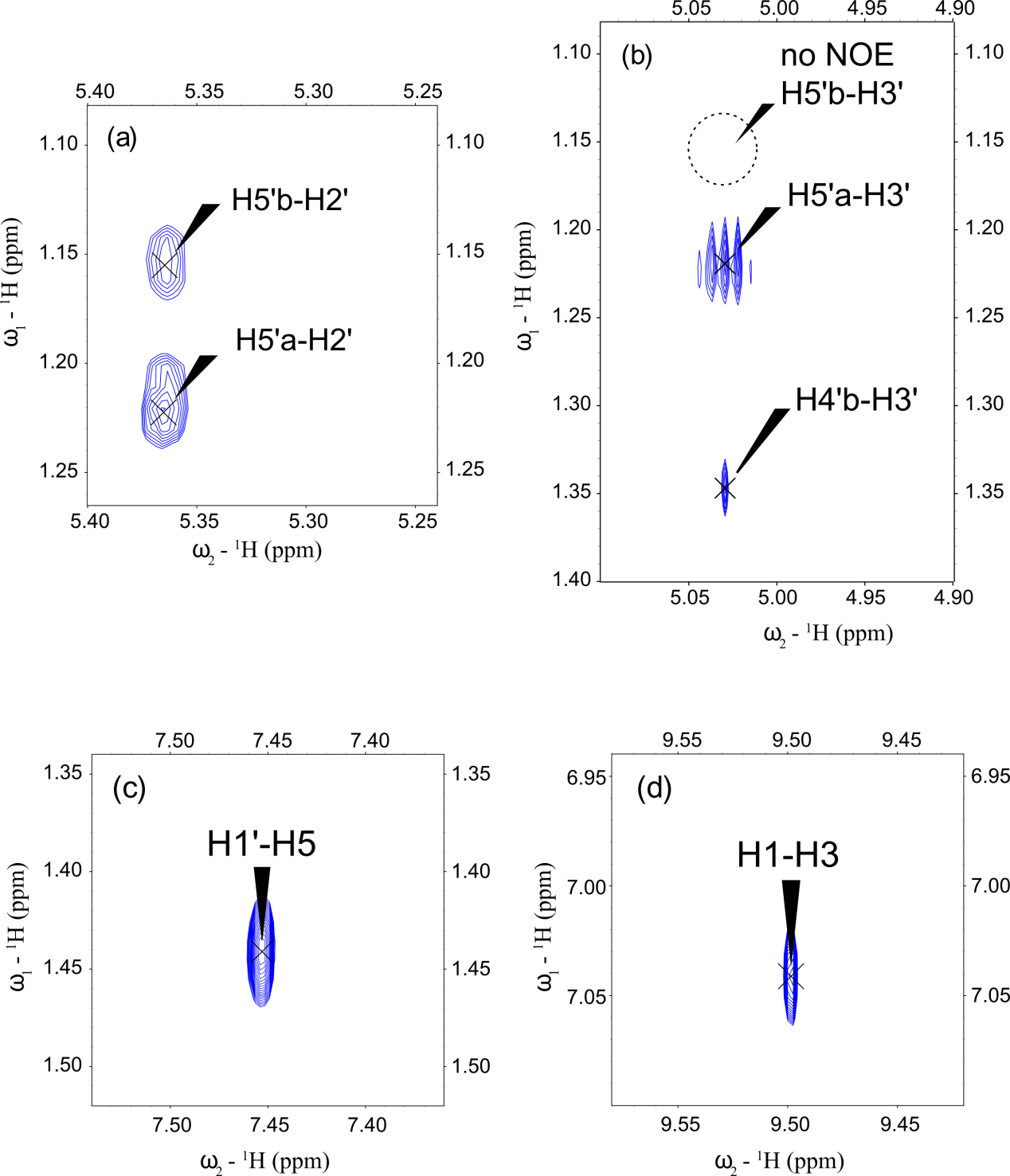


**Figure S6.** Selected regions of NOESY spectrum of fusariumin A. The mixing time of the NOESY spectrum is 200 ms.


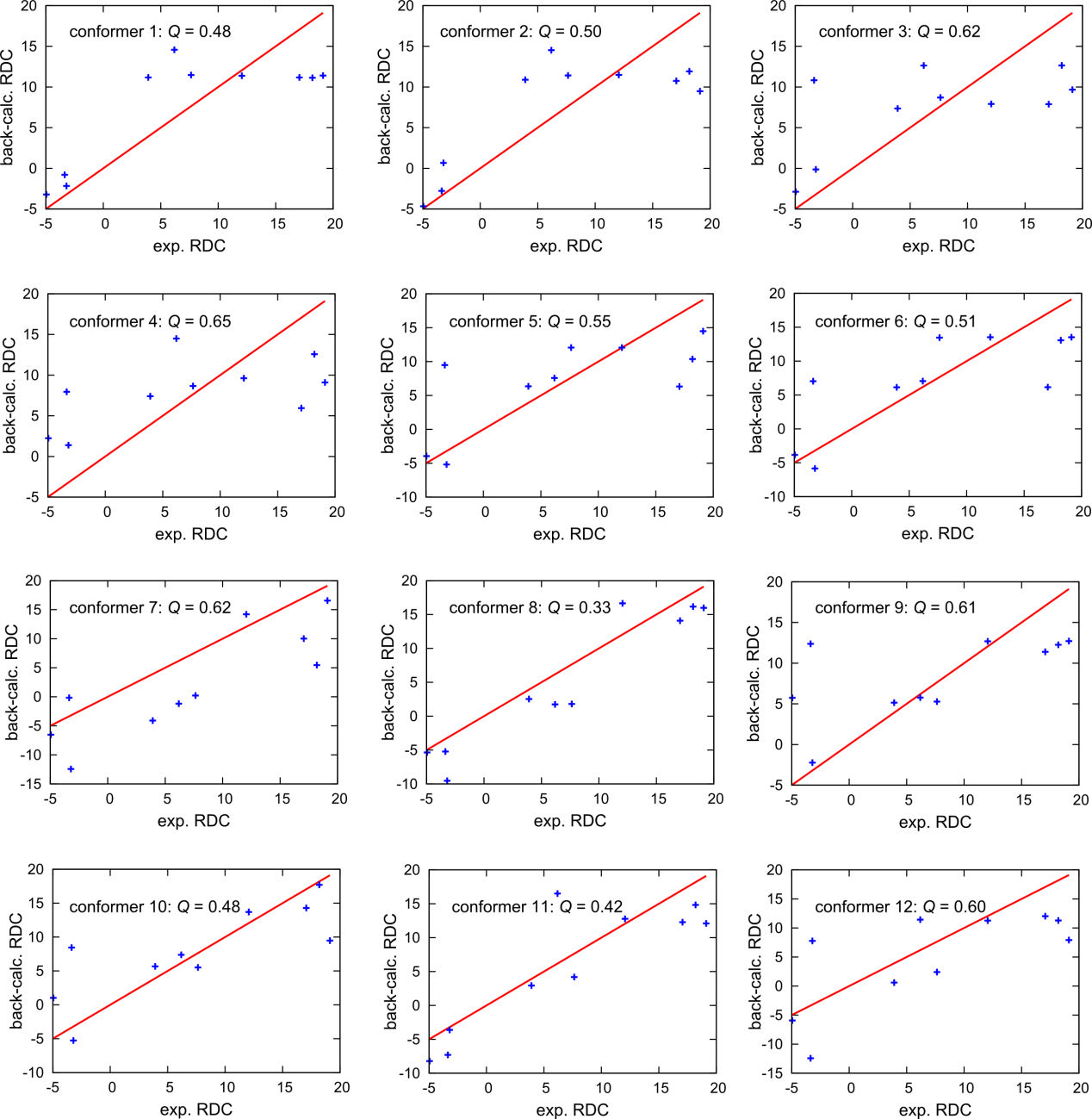


**Figure S7.** Comparison of the correlations of experimental and back-calculated RDCs

of 12 possible conformers of fusariumin A with the configuration (2'*R*,3'*S*) or (2'*S*,3'*R*).


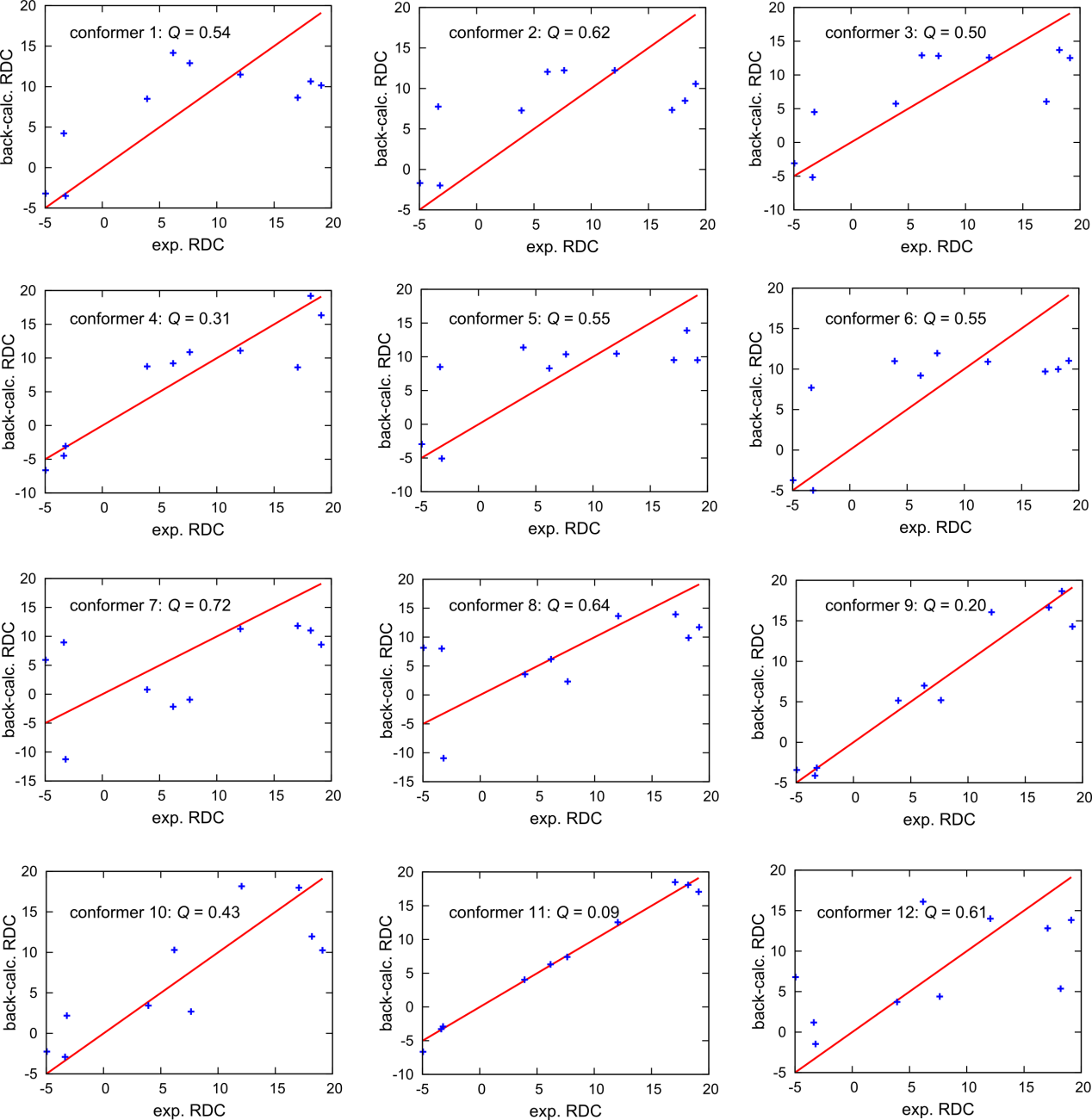


**Figure S8.** Comparison of the correlations of experimental and back-calculated RDCs

of 12 possible conformers of fusariumin A with the configuration (2'*R*,3'*R*) or (2'*S*,3'*S*).


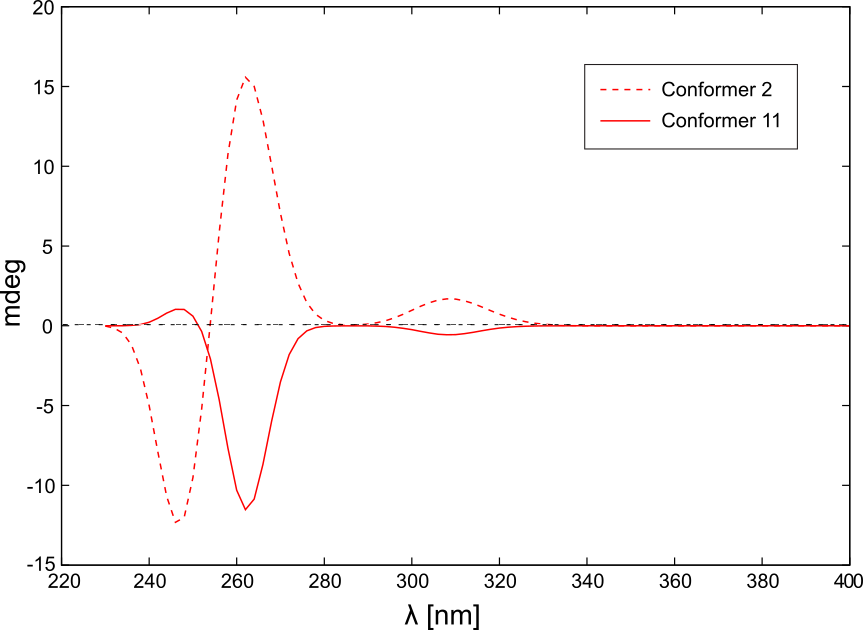


**Figure S9.** Comparison of calculated ECD spectra between RDC determined conformer 11 and conformer 2 which was identified by DFT alone to have the lowest energy but was in disagreement with the NMR experimental data. All the conformers have a (2'*R*,3'*R*) configuration.


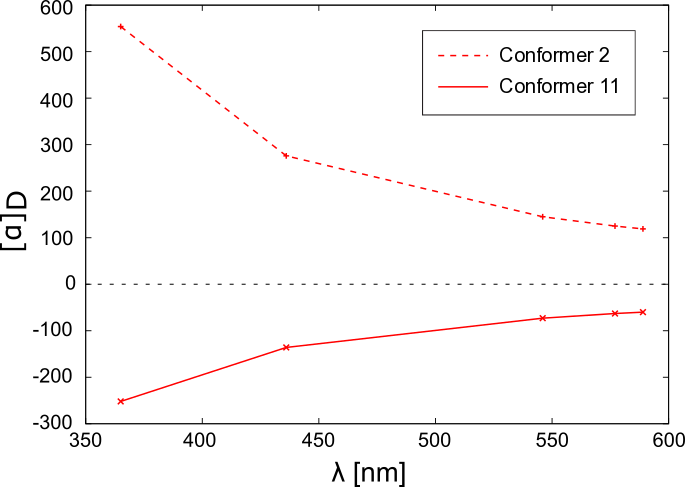


**Figure S10.** Comparison of calculated ORD spectra between RDC determined conformer 11 and conformer 2 which was identified by DFT alone to have the lowest energy but was in disagreement with the NMR experimental data. All the conformers have a (2'*R*,3'*R*) configuration.

**Table S2.** Important dihedral angles (deg) of possible conformers 1-12 for both possible relative configurations of fusariumin A. All structures were optimized with DFT at the B3LYP/6-31G(d) level using the IEFPCM solvent continuum model with DMSO as the solvent.

| **Configuration** | **Conformer** | **H2**'**-C2**'**-N-C5** | **H3**'**-C3**'**-C2**'**-H2**' | **O-C3**'**-C4**'**-C5**' |
| --- | --- | --- | --- | --- |
|  |  |  |  |  |
| (2'*S*,3'*S*)  or  (2'*R*,3'*R*) | 1 | -173.5 | 60.9 | -65.9 |
|  | 2 | 11.7 | 63.1 | -64.5 |
|  | 3 | -150.7 | -58.5 | -66.9 |
|  | 4 | 18.6 | -66.2 | -65.9 |
|  | 5 | -175.6 | -173.3 | -58.6 |
|  | 6 | 0.5 | -169.8 | -59.5 |
|  | 7 | -177.4 | -170.9 | -145.7 |
|  | 8 | 0.4 | -170.0 | -146.8 |
|  | 9 | -141.5 | -59.5 | -161.3 |
|  | 10 | 17.5 | -58.8 | -146.5 |
|  | 11 | 177.6 | 60.3 | -171.2 |
|  | 12 | 12.9 | 67.6 | -168.4 |
| (2'*S*,3'*R*)  or  (2'*R*,3'*S*) | 1 | -17.0 | -62.1 | -64.1 |
|  | 2 | 156.6 | -63.8 | -64.8 |
|  | 3 | 1.7 | -173.2 | -62.7 |
|  | 4 | 174.8 | -178.7 | -61.5 |
|  | 5 | -17.5 | 67.9 | -66.8 |
|  | 6 | 151.2 | 63.5 | -66.9 |
|  | 7 | -16.6 | 67.2 | -177.1 |
|  | 8 | 154.5 | 61.9 | -179.5 |
|  | 9 | 1.3 | -177.0 | -161.2 |
|  | 10 | 171.6 | -177.3 | -152.2 |
|  | 11 | -16.5 | -60.7 | -155.0 |
|  | 12 | 164.6 | -62.5 | -162.0 |
|  |  |  |  |  |

**Table S3.** Experimental residual dipolar couplings of fusariumin A in PH-gel/DMSO together with the back-calcuated ones for the best fitting conformer 11 of (2'*R*,3'*R*) or (2'*S*,3'*S*).

| **CH-vector** | **exp. ^1^*D*_CH_ [Hz]** | **calc. ^1^*D*_CH_ [Hz]** |
| --- | --- | --- |
|  |  |  |
| C5'-H5'a | 17.1 | 18.5 |
| C5'-H5'b | 7.6 | 7.4 |
| C4'-H4'a | 3.6 | 4.0 |
| C4'-H4'b | 12.1 | 12.5 |
| C3'-H3' | 19.1 | 17.1 |
| C2'-H2' | -3.4 | -3.3 |
| C1'-H1' | -5.0 | -6.6 |
| C5-H5 | 18.2 | 18.1 |
| C4-H4 | -3.2 | -2.9 |
| C3-H3 | 6.2 | 6.3 |
|  |  |  |

**Table S4.** Comparison of the NOE integrals and the interproton distances for the best RDC-fitting conformer 11. The NOESY spectrum was acquired with a mixing time of 200 ms. The distance to the power of minus six is referenced to proton pair H5'b-H2'.

| **Atoms** | **NOE integrals** | **Distance (Å)** | **(Distance)^-6^**  **(Referenced to H5**'**b-H2**'**)** |
| --- | --- | --- | --- |
|  |  |  |  |
| H3'-H5'a | 5.60×10^5^ | 2.53 | 0.56 |
| H1'-H5 | 7.15×10^5^ | 2.61 | 0.47 |
| H3-H1 | 2.10×10^5^ | 2.55 | 0.54 |
| H5'b-H2 | 1.11×10^5^ | 2.30 | 1.00 |
| H5'a-H2 | 1.40×10^5^ | 3.11 | 0.16 |
| H4'b-H3' | 1.81×10^5^ | 3.06 | 0.18 |
| H4'a-H3' | 4.97×10^5^ | 2.50 | 0.61 |
| H2'-H1 | 3.20×10^4^ | 3.73 | 0.05 |
|  |  |  |  |

**Table S5.** Relative energy and relative Gibbs free energy of the 12 possible conformers of fusariumin A with the configuration (2'*R*,3'*R*) or (2'*S*,3'*S*). The DFT calculations were performed at the B3LYP/6-31G(d) level using the IEFPCM solvent continuum model with DMSO as the solvent. All computations were performed at 298 K and 1 atm.

| **Conformer** | **ΔE (kcal*mol^-1^)** | **ΔG (kcal*mol^-1^)** |
| --- | --- | --- |
|  |  |  |
| 1 | 3.8 | 4.2 |
| 2 | 0.0 | 0.0 |
| 3 | 1.8 | 2.6 |
| 4 | 4.9 | 5.0 |
| 5 | 0.9 | 0.4 |
| 6 | 1.4 | 2.3 |
| 7 | 2.9 | 3.4 |
| 8 | 3.4 | 4.6 |
| 9 | 3.7 | 4.1 |
| 10 | 7.8 | 8.8 |
| 11 | 0.4 | 1.1 |
| 12 | 4.9 | 5.5 |
|  |  |  |
